# Death of the Discoverer of Fœtal Auscultation

**Published:** 1878-05

**Authors:** 


Death of the Discoverer of Fœtal Auscultation.—The Count de Kergaredec, the first to apply auscultation to the detection of the fœtal heart in pregnancy, died lately in Paris at an advanced age. His son in announcing his death to the French Academy said: "Among his children who stood around his death-bed was that beloved daughter, the beating of whose heart her father heard whilst she was still in her mother's womb."—*The Doctor.*

